# Silent Signals: Analyzing Repolarization Heterogeneity and Autonomic Modulation in Children With Euthyroid Hashimoto’s Thyroiditis

**DOI:** 10.7759/cureus.80925

**Published:** 2025-03-20

**Authors:** Ozlem Turan, Tugba Burcu Ozturk Gomec

**Affiliations:** 1 Pediatric Cardiology, Antalya Training and Research Hospital, University of Health Sciences, Antalya, TUR

**Keywords:** autoimmune thyroid disease, autonomic nervous system dysfunction, cardiac thyroid, euthyroid, hashimoto's thyroiditis, new-onset arrhythmia

## Abstract

Background: Hashimoto's thyroiditis is the most prevalent autoimmune thyroid condition in children, typically appearing in a euthyroid state. The cardiovascular impact of euthyroid Hashimoto’s thyroiditis (eHT) remains poorly understood, especially regarding its connection to arrhythmias and autonomic dysfunction. This study aimed to evaluate electrocardiographic markers of repolarization inhomogeneity (P-wave dispersion (Pd), QT dispersion (QTd), and peak-to-end interval of the T-wave (Tp-e)) as well as heart rate variability (HRV) in children with eHT to assess the asymptomatic cardiovascular effects that may predispose them to future arrhythmias.

Materials and methods: This retrospective analysis involved 67 patients and 50 healthy controls. Patients were evaluated for anthropometric and biochemical parameters, electrocardiogram (ECG) results, echocardiographic findings, and 24-hour Holter monitoring. ECG parameters were measured and analyzed for repolarization indices, while HRV was assessed using time-domain and frequency-domain analyses.

Results: Patients exhibited significantly longer P max, Pd, QT intervals, QTd, and Tp-e intervals than the controls (all p < 0.001). A receiver operating characteristic analysis indicated that a Pd of 51 ms predicted the disease with 67% sensitivity and 72% specificity (area under the curve: 0.733, p = 0.001, 95% CI: 0.643-0.823). Time-domain HRV parameters (SD of all normal-to-normal intervals and SD of all the five-minute normal-to-normal intervals) were significantly lower in eHT patients, indicating an autonomic imbalance. At the same time, frequency-domain analyses revealed no significant differences.

Conclusions: Children with eHT exhibit increased myocardial repolarization heterogeneity and reduced HRV, indicating a potential risk for arrhythmias. These findings highlight the necessity of regular cardiac symptom inquiry and ECG controls in the follow-up of these patients, considering both thyroid function and underlying autoimmune processes. Further research is needed to clarify the long-term cardiovascular risks of eHT in children.

## Introduction

Hashimoto's thyroiditis is the most prevalent chronic autoimmune thyroid disorder in children [1]. The clinical presentation can vary widely, ranging from completely asymptomatic to severe symptoms of thyroid dysfunction. The euthyroid state is the most prevalent presentation in children [2-4]. Thyroid hormones affect the cardiovascular system, playing a crucial role in maintaining a normal heart rhythm and potentially contributing to arrhythmias [5]. It is well-documented that thyroid dysfunction is linked to cardiovascular morbidity and mortality. However, the cardiovascular effects of euthyroid Hashimoto’s thyroiditis (eHT) are not yet fully understood [6]. Some studies suggest that transmural dispersion of repolarization is a significant factor in the development of arrhythmias. Electrocardiographic markers such as P-wave dispersion (Pd), QT dispersion (QTd), the peak-to-end interval of the T-wave (Tp-e), and the Tp-e/QT ratio are well-recognized for assessing myocardial repolarization. Elevated levels of repolarization parameters are associated with an increased risk of arrhythmias [7-10].

Heart rate variability (HRV) is a reproducible, noninvasive electrocardiographic method for assessing the impact of the sympathetic and parasympathetic components of the autonomic nervous system on the sinus node [11,12]. Reduced HRV is closely linked to sympathovagal imbalance, which is characterized by heightened sympathetic activity and reduced vagal tone [6,9,10]. It is a strong predictor of arrhythmias and sudden cardiac death [9,13]. Recent studies have demonstrated a significant link between autonomic imbalance and cardiovascular diseases in patients with thyroid dysfunction [9,13].

While there is considerable knowledge about the cardiovascular effects of both clinical and subclinical thyroid disorders, a comprehensive understanding of the relationship between eHT and conduction abnormalities in children is still lacking. This study investigated Pd, QTd, and their related components as indicators of repolarization inhomogeneity in children with eHT, which have not previously been evaluated in these patients. We also analyzed HRV to assess cardiac autonomic modulation in this patient group. This research aimed to identify asymptomatic cardiovascular impacts associated with arrhythmias, ultimately seeking to reduce the risk of future cardiovascular events.

## Materials and methods

Study design and population

Using a computerized database, this retrospective study analyzed 67 patients (14 males) and 50 control subjects (18 males). The patient cohort included individuals diagnosed with eHT evaluated in the pediatric cardiology department for palpitations. The inclusion criteria stipulated that patients must have been diagnosed with eHT for over six months to prevent misdiagnosis and ensure euthyroid status, normal thyroid function, and good metabolic control. Patients were excluded if they had any additional autoimmune disorders, liver or kidney dysfunction, hypertension, systemic infections, obesity (body mass index (BMI, kg/m²) ≥ ±2 SD was used for obesity), dyslipidemia, congenital heart defects, arrhythmias, electrolyte disturbances, anemia, or a history of thyroid hormone replacement therapy. The control group comprised healthy children matched by age and gender who were assessed for palpitations.

Anthropometric parameters were assessed in all patients. Body surface area (BSA, m²) and BMI (kg/m²) were calculated. Biochemical parameters, including serum autoantibody levels, thyroid-stimulating hormone (TSH), free triiodothyronine (fT3), free tetraiodothyronine (fT4), and serum electrolytes, were reviewed from the hospital database. We analyzed results from systolic and diastolic blood pressure (BP) measurements, electrocardiograms (ECG), echocardiograms, and 24-hour Holter monitoring for all patients. All recordings were evaluated by a single investigator blinded to the participants’ identities, thereby eliminating inter-observer variability. The study was conducted following the ethical standards outlined in the 1964 Declaration of Helsinki, and the protocol was approved by the Institutional Ethics Committee of Antalya Training and Research Hospital (approval number: 3/8, approval date: March 21, 2024).

Electrocardiographic measurements

The 12-lead ECG data was reviewed by enlarging it on a computer screen, with speeds set to 25 mm/s and 50 mm/s and an amplitude of 10 mm/mV. The heart rate was determined by assessing the RR interval from the tracings.

P-Wave Dispersion

P-wave duration refers to the time interval between the beginning and end of a P-wave. The P-wave's intersection defines the P-wave's start and end points with the isoelectric line and the point where the P-wave ends at the isoelectric line, respectively. Pd was calculated as the difference between P max and P min. Acceptable electrocardiography was determined by the ability to measure P-wave duration in at least eight simultaneously recorded ECG leads [14,15].

QT-Wave Dispersion

QT is the time interval from the start of the QRS complex to the end of the T-wave when it returns to the isoelectric line. In the presence of U-waves, the end of the T-wave is marked by the nadir between the T- and U-waves. If the end of the T-wave cannot be determined, that lead is excluded. Three consecutive QT intervals are measured, and the average is calculated for each lead. The QTc duration is calculated using Bazett’s formula (QTc = QT/√RR). QTd is the difference between the maximum and minimum QT intervals recorded on the ECG. The Tp-e interval refers to the time between the peak of the T-wave and the end of the T-wave on the electrocardiogram. For the Tp-e interval, measurements are taken from the precordial leads, with the longest Tp-e interval recorded. Tp-e/QTc interval ratios are calculated based on these measurements [7,16].

Echocardiographic examinations

Echocardiographic evaluations were performed on all participants using a Philips Affinity 50 Cardiac Ultrasound with a 5-1 MHz transducer (Bothell, WA, USA). Two-dimensional and M-mode echocardiography was used to measure the left ventricular end-diastolic dimension, left ventricular end-systolic dimension, left ventricular end-diastolic posterior wall thickness, left ventricular ejection fraction (EF), and left ventricular fractional shortening (FS), following the guidelines set by the American Society of Echocardiography [17].

24-hour Holter monitoring

The 24-hour Holter recordings were performed using a three-channel DMS 300-3A Holter system (DM Software, Inc., Stateline, NV, USA). HRV analysis focused on the variations in consecutive RR intervals, with measurements taken continuously throughout the 24-hour period. Abnormal beats and segments containing artifacts were excluded from the analysis. Recordings shorter than 18 hours were not considered to avoid the impact of circadian variations on HRV. HRV was evaluated by calculating time- and frequency-domain indices from the 24-hour data. HR variables were analyzed using normal-to-normal intervals following the guidelines established by the Task Force of the European Society of Cardiology and the American Society of Pacing and Electrophysiology [12].

Time-Domain Variables

The time-domain indices of HRV were evaluated for the following metrics: SD of all normal-to-normal intervals (SDNN), mean of the SD of all normal-to-normal intervals for each five-minute segment (SDNNI), SD of all the five-minute normal-to-normal intervals (SDANN), square root of the mean of the squared differences between adjacent normal-to-normal intervals (rMSSD), and percentage of the differences between adjacent normal-to-normal intervals greater than 50 milliseconds (PNN50). While rMSSD and PNN50 primarily reflect parasympathetically mediated heart rate changes, the other time-domain measures represent a combination of parasympathetic, sympathetic, and various physiological influences.

Frequency-Domain Variables

Spectral power was calculated across three frequency regions of interest: very-low-frequency (VLF) index (0.017-0.05 Hz), low-frequency (LF) index (0.05-0.15 Hz), and high-frequency (HF) index (0.15-0.50 Hz). Additionally, we measured the total power (covering all frequencies above 0.017 Hz) and the LF/HF ratio. HF reflects cardiac vagal activity, while the vagal and sympathetic systems influence LF. The LF/HF ratio is a key metric for assessing autonomic balance.

Statistical analysis

Statistical Analysis System version 9.4 (SAS Institute, Cary, NC, USA) was utilized to analyze the data collected in this study. All data were presented as the mean and SD or median (IQR) for continuous variables, while categorical variables were represented as percentages, depending on the distribution's normality. This study's dependent and independent variables underwent a normality assessment using the Kolmogorov-Smirnov test. Additionally, skewness values were examined. Further analysis was conducted as follows: an independent group t-test was employed to identify differences between the two groups; Pearson correlation analysis was performed to explore the relationship between quantitative variables; Chi-square analysis was conducted to assess relationships among qualitative variables; and receiver operating characteristic (ROC) analysis was used to evaluate the sensitivity and specificity of delta Pd. A significance level of 0.05 was established throughout this study.

## Results

The mean age was 12.7 ± 3.19 years (range: 7.1-17.0 years) in the patient group, compared to 12.5 ± 2.69 years (range: 7.3-17.5 years) in the control group (p = 0.695). HR and BP measurements were comparable between the groups. Patients with eHT exhibited significantly higher thyroglobulin and thyroid peroxidase antibody levels (p < 0.001). No notable differences in fT3, fT4, and TSH levels were found between the groups.

The ECG findings revealed that 16 patients (13.6%) had sinus arrhythmia, while eight (6.8%) presented with an incomplete right bundle branch block. Additionally, three (2.5%) patients experienced ventricular extrasystoles on their ECG, and Holter monitoring indicated only rare ventricular extrasystoles without any pairs or runs. The conventional echocardiographic evaluation showed that parameters such as left ventricular end-diastolic diameter, interventricular septum diastolic thickness, left ventricular posterior wall diastolic thickness, FS, and EF in patients with eHT were not significantly different from those in the control group (all p > 0.05). Mild mitral regurgitation was observed in four (3.4%) patients and three (2.5%) controls (Table 1).

**Table 1:** Demographic characteristics, laboratory results, and conventional echocardiographic findings in the study groups eHT: euthyroid Hashimoto's thyroiditis, BSA: body surface area, BMI: body mass index, SBP: systolic blood pressure, DBP: diastolic blood pressure, HR: heart rate, Tg-Ab: thyroglobulin antibody, TPO-Ab: thyroid peroxidase antibody, TSH: thyroid-stimulating hormone, fT3: free triiodothyronine, fT4: free tetraiodothyronine, EF: ejection fraction, FS: fractional shortening, LVDd: left ventricular end-diastolic diameter, IVSd: interventricular septum diastolic thickness, LVPWd: left ventricular posterior wall diastolic thickness

	eHT (n = 67)	Controls (n = 50)	p-value
Age (years)	12.7 ± 3.19	12.5 ± 2.69	0.695
Female/male	53/14	32/18	0.069
Weight (kg)	50.6 ± 18.65	46.8 ± 12.58	0.282
Height (cm)	151.8 ± 16.61	152.0 ±14.89	0.969
BSA (m^2^)	1.3 ± 0.9	1.2 ± 0.9	0.781
BMI (kg/m^2^)	20.1 ± 3.1	20.3 ± 2.9	0.511
SBP (mmHg)	110 ± 8.9	106 ± 8.5	0.255
DBP (mmHg)	69 ± 7.9	66 ± 7.1	0.368
HR (beats/min)	85.1 ± 11.43	85.3 ± 11.72	0.923
Tg-Ab (IU/mL)	128.3 ± 114.49	1.9 ± 0.72	<0.001
TPO-Ab (IU/mL)	284.6 ± 283.37	2.4 ± 0.68	<0.001
TSH (mIU/L)	2.7 ± 1.00	2.6 ± 0.95	0.713
fT3 (pg/mL)	0.99 ± 0.6	1.1 ± 0.3	0.875
fT4 (pg/mL)	1.0 ± 0.21	1.2 ± 1.17	0.179
EF (%)	69.1 ± 3.86	68.8 ± 3.98	0.803
FS (%)	37.7 ± 3.69	37.2 ± 3.67	0.499
LVDd (mm)	38.8 ± 4.52	39.2 ± 3.92	0.588
IVSd (mm)	7.2 ± 0.44	7.2 ± 0.42	0.761
LVPWd (mm)	7.3 ± 0.41	7.3 ± 0.40	0.955

In the eHT and control groups, P max was 100.9 ± 5.89 ms and 95.0 ± 6.54 ms, respectively (p < 0.001); P min was 48.5 ± 5.77 ms and 49.3 ± 5.39 ms, respectively (p = 0.382); QT max was 327.8 ± 11.91 ms and 316.7 ± 13.57 ms, respectively (p < 0.001); QT min was 296.9 ± 11.99 ms and 297.6 ± 13.70 ms, respectively (p = 0.634); QTc max was 389.3 ± 29.22 ms and 376.7 ± 29.62 ms, respectively (p = 0.048); and QTc min was 352.8 ± 28.72 ms and 354.1 ± 28.88 ms, respectively (p = 0.768). Pd, QTd, corrected QTd (QTcd), Tp-e max, Tp-e, Tp-e/QT, and Tp-e/QTc were significantly higher in the eHT group compared to the control group. All repolarization parameters are summarized in Table 2.

**Table 2:** Electrocardiographic repolarization parameters of patients with eHT and the control group P max: maximum P-wave interval, P min: minimum P-wave interval, Pd: P-wave dispersion, QT max: maximum QT interval, QT min: minimum QT interval, QTd: QT dispersion, QTc max: corrected QT maximum interval, QTc min: corrected QTc minimum interval, QTcd: corrected QT dispersion

	eHT (n = 67)	Controls (n = 50)	p-value
Pd (ms)	51.9 ± 5.33	47.2 ± 5.32	<0.001
QTd (ms)	31.0 ± 9.98	19.0 ± 8.24	<0.001
QTcd (ms)	36.6 ± 12.15	22.6 ± 9.80	<0.001
Tp-e (ms)	58.7 ± 5.31	53.0 ± 4.41	<0.001
Tp-e/QT	0.2 ± 0.02	0.2 ± 0.01	0.001
Tp-e/QTc	0.2 ± 0.02	0.1 ± 0.02	0.003

**Figure 1:**
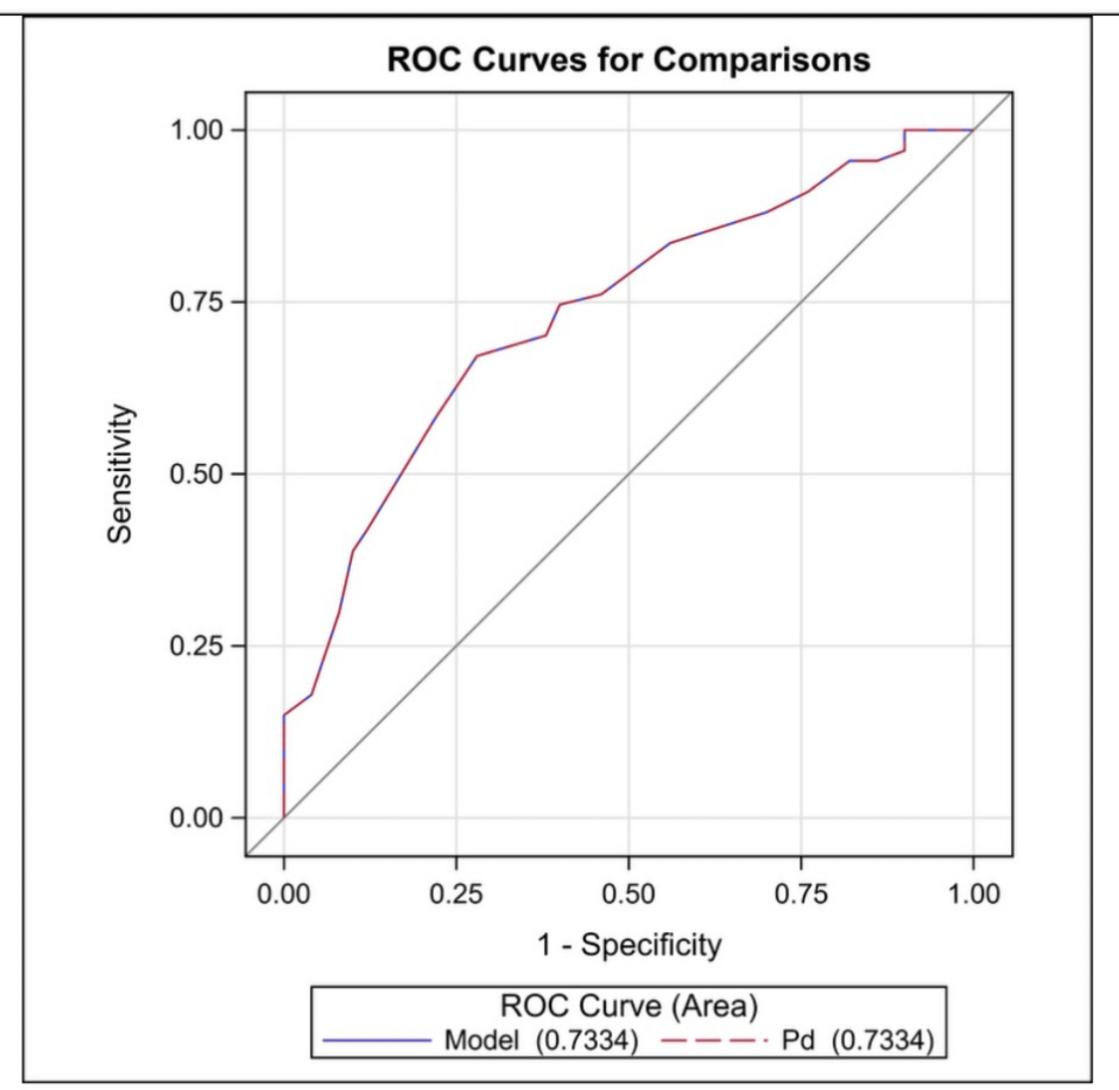
ROC curve analysis of Pd for predicting euthyroid hypothyroidism ROC: receiver operating characteristic, Pd: P-wave dispersion

The ROC curve analysis indicated that a Pd value of 51 ms predicted euthyroid hypothyroidism with 67% sensitivity and 72% specificity (area under the curve: 0.733, p = 0.001, 95% CI: 0.643-0.823) (Figure 1).

The 24-hour Holter monitoring indicated that the minimum, maximum, and average heart rates were not significantly different between the groups (p = 0.241, p = 0.128, and p = 0.154, respectively). A comparison of HRV parameters was conducted for both time and frequency domains. In the time-domain parameters, SDNN and SDANN were significantly lower in children with eHT compared to the control group (p = 0.003, p = 0.012, respectively). However, other time-domain parameters, such as RMSSD and pNN50, did not reveal significant differences between the groups. Additionally, frequency-domain analyses did not demonstrate significant differences between the patient and control groups (Table 3).

**Table 3:** Comparison of the time- and frequency-domain HRV parameters between the patient and control groups SDNN: standard deviation of all normal-to-normal intervals, SDANN: standard deviation of all five-minute normal-to-normal intervals, rMSSD: square root of the mean of the squared differences between adjacent normal-to-normal intervals, PNN50: percentage of the difference between adjacent normal-to-normal intervals greater than 50 milliseconds, VLF: very low frequency, LF: low frequency, HF: high frequency, LF/HF: low frequency/high frequency

	Patients (n = 67)	Controls (n = 50)	p-value
Max HR (min^−1^)	171.2 ± 15	176.5 ± 13.5	0.128
Min HR (min^−1^)	48.7 ± 7	51.1 ± 6.9	0.159
Average HR (min^−1^)	86.9 ± 10.5	90.9 ± 8.5	0.090
Time-domain parameters			
SDNN (ms)	114.5 ± 26.8	137.1 ± 23.5	0.003
SDNN index (ms)	63.5 ± 19.7	67.8 ± 18.1	0.750
SDANN (ms)	104.3 ± 31.4	127 ± 31.7	0.012
rMSSD (ms)	43 ± 22	46 ± 23	0.660
PNN50 (%)	19.4 ± 10.7	22.1 ± 10.3	0.102
Total power (ms^2^)	3771 (2358.4-5102.3)	3805 (2542.2-5810.9)	0.595
Frequency-domain parameters			
VLF (ms^2^)	2192 (1324.3-3197.4)	2167.2 (1543.3-3237.4)	0.620
LF (ms^2^)	748.3 (430.5-950.5)	761 (423.2-1050.5)	0.520
HF (ms^2^)	668.1 ± 293.3	757.1 ± 384.1	0.210
LF/HF	1.6 (0.9-2.8)	1.4 (0.9-5.9)	0.813

## Discussion

While there is considerable knowledge about the cardiovascular effects of thyroid disorders, our understanding of how autoimmune euthyroid chronic thyroiditis affects the cardiovascular system remains limited. This study provides pediatric data indicating that the transmural dispersion of the repolarization indices (Pd, QTc max, QTd, Tp-e interval, and Tp-e/QTc ratio) statistically increased in eHT patients compared to healthy controls. Additionally, HRV parameters were reduced in the eHT group, suggesting a sympathovagal imbalance in these patients. These findings emphasize heightened repolarization heterogeneity and autonomic dysfunction, possibly leading to arrhythmias in children with eHT.

Thyroid hormones target the cardiovascular system. Patients with hypothyroidism experience decreased binding to the receptors on myocytes and decreased catecholamine sensitivity in vascular structures. This condition is often associated with various electrophysiological alterations, decreased cardiac contractility, and reduced cardiac output [18,19]. Numerous studies about ventricular repolarization related to thyroid dysfunction have recently been published [7-10,16,20].

Impaired atrial conduction plays a critical role in the pathophysiology of atrial arrhythmias, particularly atrial fibrillation (AF) [21,22]. Pd is a significant marker for assessing intra-atrial and inter-atrial conduction times. An elevated Pd suggests a delayed and inhomogeneous conduction of sinus impulses, a well-established characteristic of atrial arrhythmias [14,15]. A Pd value of ≥40 ms has been linked to an increased risk of AF [22]. Irdem et al. found that P max and Pd were extended in patients with subclinical hypothyroidism [23]. Additionally, a systematic review of adult populations showed that subclinical hypothyroidism increases the risk of AF [24]. While the connection between atrial arrhythmias and thyroid hormones is well-established, evidence regarding euthyroid states remains scarce [6]. Consistent with the literature, we demonstrated that P max and Pd were significantly higher than in the control group, despite having normal thyroid hormone levels. The ROC curve analysis indicated that a Pd value of 49.5 ms predicted euthyroid hypothyroidism with 66.7% sensitivity and 62.1% specificity.

QTd indicates the inhomogeneity of ventricular repolarization. The results of studies have demonstrated that an increased QTd is correlated with an increased risk of ventricular arrhythmias [7-10,25]. Galetta et al. conducted the initial study demonstrating the association between subclinical hypothyroidism and increased QTd. They identified an increased QTd in a cohort of 42 adults with subclinical hypothyroidism, revealing a positive correlation between QTd, QTcd, and TSH levels. Notably, these alterations improved following L-thyroxine treatment [10]. Subsequent studies have reported significant elevations in both QTd and QTcd linked to TSH levels, reinforcing these findings [8,10,20]. Similarly, we observed a marked increase in QTd and QTcd among eHT children compared to healthy controls.

One of the emerging markers of ventricular repolarization that has gained attention in recent years is the Tp-e interval. The Tp-e/QT and Tp-e/QTc ratios are increasingly recognized as valuable parameters for predicting arrhythmias [7,8,16,26]. Gurdal et al. compared the Tp-e/QT ratio between patients with subclinical hypothyroidism and a control group, revealing statistically significant differences [8]. Similarly, other studies have found a notable correlation between the Tp-e interval, the Tp-e/QT, and the Tp-e/QTc and serum TSH levels, indicating that thyroid function may influence these markers [7,16]. Our findings align with these studies, supporting that Tp-e metrics could serve as important indicators of repolarization abnormalities and potential cardiovascular risks, especially in populations with thyroid dysfunction.

In hypothyroidism, alterations in cardiac autonomic function may arise at the hypothalamic level. Numerous studies have suggested that TSH is critical in establishing sympathovagal imbalance [27,28]. Meta-analyses have highlighted significant correlations between TSH levels and HRV measures, indicating that TSH changes can impact autonomic regulation [28]. Additionally, a negative association has been reported between serum TSH levels and time-domain HRV parameters in patients with subclinical hypothyroidism [10,13].

Time-domain measures like SDNN and SDANN reflect a greater influence of the sympathetic nervous system on HRV, while lower frequency-domain LF values suggest an increase in sympathetic activity. In contrast, reduced HF values indicate decreased vagally mediated changes and lower time-domain parameters such as rMSSD and PNN50. The LF/HF ratio measures sympathovagal balance, indicating an overall increase in sympathetic activity [9]. Our study found that SDNN and SDANN were significantly lower in patients with eHT, indicating autonomic nervous system imbalances, decreased HRV, increased sympathetic tone, and a loss of parasympathetic tone. In line with our findings, Kilic et al. reported reduced time-domain parameters in children with eHT despite no significant TSH elevation, implying the involvement of other pathogenic factors. They emphasized the role of vascular endothelial cells in cardiovascular regulation through the secretion of various vasoactive compounds, such as nitric oxide and prostacyclin, which can influence autonomic function [29]. Moreover, Akgul et al. documented a significant decrease in HRV among eHT patients, supporting the notion that HRV can be altered without overt thyroid dysfunction [6]. This implies that the mechanisms underlying cardiac autonomic and functional changes in eHT may be linked to abnormal cytokine profiles, potentially impacting cardiovascular health [6,30].

Hashimoto's thyroiditis is the most prevalent autoimmune thyroid disease in children. Many patients are euthyroid at diagnosis; however, the underlying mechanisms responsible for cardiovascular effects remain partially understood [1-3]. The majority of euthyroid patients experience a gradual, progressive decline in thyroid function. It is well-recognized that most of these patients will eventually develop hypothyroidism. Consequently, one may hypothesize that the gradual progression of thyroid dysfunction in Hashimoto’s thyroiditis contributes to adverse cardiovascular effects [7,8,20,30]. Even without thyroid hormone deficiency, eHT patients experience cardiovascular alterations and autonomic dysfunction due to chronic autoimmune activation, inflammation, endothelial dysfunction, and cytokine-mediated immune dysregulation [6,13,29]. This indicates that the autoimmune nature of the condition, rather than just the influence of circulating thyroid hormones, may underlie the cardiovascular changes observed. These mechanisms may contribute to subtle but significant cardiovascular risks, highlighting the need for early monitoring and preventive strategies in eHT patients. This manifests as reduced HRV, suppressed vagal tone, and increased sympathetic activity. While eHT aligns with subclinical hypothyroidism in terms of autonomic dysfunction and cardiovascular risk, it appears to have a less severe impact on repolarization and HRV, possibly due to preserved thyroid hormone function. However, the autoimmune component in eHT suggests a distinct pathway that warrants further investigation [13]. Our findings are consistent with previous studies showing that some chronic inflammatory conditions, including systemic lupus erythematosus, rheumatoid arthritis, psoriasis, and sarcoidosis, are associated with decreased HRV and increased heterogeneity in myocardial repolarization [26]. It’s also essential to recognize that the clinical presentations of HT can evolve. Consequently, patients with eHT may have previously experienced symptoms of hypothyroidism or hyperthyroidism, despite appearing euthyroid during the assessment. This highlights the complexity of managing thyroid dysfunction and its potential cardiovascular consequences in affected individuals.

Although there is no universal guideline for cardiovascular monitoring in pediatric eHT patients, evidence suggests that regular follow-up with cardiac autonomic function assessments, ECG, and inflammation markers may help early identification of individuals at risk. The lack of standardized criteria for repolarization abnormalities in eHT makes it difficult to assess cardiovascular risks accurately. Addressing this issue requires meta-analyses to identify consistent trends in QTd and HRV changes; standardized ECG and HRV protocols to improve measurement consistency; long-term studies to determine whether these abnormalities lead to clinical events; and research on inflammation and autoimmunity to clarify their role in repolarization disturbances [13,29].

Several factors, including a small sample size, retrospective design, and the lack of patient reassessment, limit the present study. In addition, although existing studies have investigated parameters such as Pd, QTd, QTcd, and Tp-e, there is no consensus on reference limits. This lack of standardization complicates the identification of pathological values, making it difficult to determine which thresholds are associated with an increased risk of cardiac disease. Consequently, the definition of clinically significant values in this context remains controversial. Also, intra-observer variability was not assessed.

## Conclusions

The findings of this study contribute to the evidence linking eHT with autonomic dysregulation and increased myocardial repolarization heterogeneity, indicating that eHT patients may face an increased risk for arrhythmias. Analyzing the "silent signals" observed in ECG may provide insights for predicting arrhythmic events. Besides thyroid hormones, potential mechanisms related to autoimmunity may help to clarify the connections between cardiac autonomic dysfunction and functional changes in chronic thyroiditis. However, the existing molecular, physiological, and clinical evidence remains inconclusive. Thus, more prospective studies with larger numbers of patients and longer follow-ups are needed to establish the definitive correlation between arrhythmias and pediatric eHT and to determine the clinical significance of the effects of these "silent signals."
